# Mycorrhizal Symbiosis and Local Adaptation in *Aster amellus*: A Field Transplant Experiment

**DOI:** 10.1371/journal.pone.0093967

**Published:** 2014-04-07

**Authors:** Hana Pánková, Jana Raabová, Zuzana Münzbergová

**Affiliations:** 1 Institute of Botany, Academy of Sciences of the Czech Republic, Průhonice, Czech Republic; 2 Department of Botany, National Museum, Prague, Czech Republic; 3 Department of Botany, Faculty of Science, Charles University, Prague, Czech Republic; Centro de Investigación y de Estudios Avanzados, Mexico

## Abstract

Many plant populations have adapted to local soil conditions. However, the role of arbuscular mycorrhizal fungi is often overlooked in this context. Only a few studies have used reciprocal transplant experiments to study the relationships between soil conditions, mycorrhizal colonisation and plant growth. Furthermore, most of the studies were conducted under controlled greenhouse conditions. However, long-term field experiments can provide more realistic insights into this issue. We conducted a five-year field reciprocal transplant experiment to study the relationships between soil conditions, arbuscular mycorrhizal fungi and plant growth in the obligate mycotrophic herb *Aster amellus*. We conducted this study in two regions in the Czech Republic that differ significantly in their soil nutrient content, namely Czech Karst (region K) and Ceske Stredohori (region S). Plants that originated from region S had significantly higher mycorrhizal colonisation than plants from region K, indicating that the percentage of mycorrhizal colonisation has a genetic basis. We found no evidence of local adaptation in *Aster amellus*. Instead, plants from region S outperformed the plants from region K in both target regions. Similarly, plants from region S showed more mycorrhizal colonisation in all cases, which was likely driven by the lower nutrient content in the soil from that region. Thus, plant aboveground biomass and mycorrhizal colonisation exhibited corresponding differences between the two target regions and regions of origin. Higher mycorrhizal colonisation in the plants from region with lower soil nutrient content (region S) in both target regions indicates that mycorrhizal colonisation is an adaptive trait. However, lower aboveground biomass in the plants with lower mycorrhizal colonisation suggests that the plants from region K are in fact maladapted by their low inherent mycorrhizal colonization. We conclude that including mycorrhizal symbiosis in local adaptation studies may increase our understanding of the mechanisms by which plants adapt to their environment.

## Introduction

Plant populations often adapt to their local environments [1], [2]. Local adaptations can be demonstrated by reciprocal transplant experiments showing that local genotypes perform better in their native environments than genotypes from other populations [3]. Theoretically, the magnitude of local adaptation should increase with increasing genetic variation within populations and with increasing environmental and phenotypic divergence between populations [2]. As a result, local adaptation is primarily expected at large spatial scales; however, it has also been found on small spatial scales (e.g., between microhabitats a few metres apart) [4].

Local adaptations in plants can often be explained by differences in the soil conditions of the studied localities [5]. While the effect of soil is most commonly explained as the effect of chemical or physical soil properties [6], [7], it may also be related to the differences in soil biota [5]. Recently, several studies have suggested that plants may be locally adapted to arbuscular mycorrhizal fungi [8]–[11]. It has been shown that plants perform better when they grow in their local soil and when they are inoculated with their native arbuscular mycorrhizal fungi [8]–[10]. In contrast, another study of 64 plant species showed that the responses of plants to both native arbuscular mycorrhizal fungi and local soil can vary largely, from negative to positive effects [11]. One study indicated that plants can change their dependence on arbuscular mycorrhizal fungi depending on the actual nutrient content of the soil [8]. Other studies suggest that the ability of plants to change the intensity of mycorrhizal colonisation is limited [9], [12].

The association of plants with arbuscular mycorrhizal fungi cannot be generally regarded as mutualism, but it can be found somewhere along the mutualism–parasitism continuum [13]. Mutualistic associations are most likely to arise in nutrient-limited environments, and parasitic associations are most likely in high-fertility environments [14]. Because the acquisition of soil resources can act as a strong selection pressure, it is likely that plants and their associated arbuscular mycorrhizal fungi exert reciprocal selective forces on each other [12]. As a result, plants, arbuscular mycorrhizal fungi, and other soil organisms are selected to their ability to coexist within local communities under ambient soil conditions (called the co-adaptation model) [12], [15]. In agreement with this theory, the effects of arbuscular mycorrhizal fungi on the number of shoots and aboveground biomass of several vascular plants have been found to vary among sites with different soil fertilities [16].

Only a few studies have used reciprocal transplant experiments to study the local adaptations of plants to soil conditions mediated by mycorrhizal fungi. Furthermore, most of the studies were performed under controlled greenhouse conditions [8]–[10], [17], [18]. Nevertheless, controlled conditions cannot include all naturally co-occurring organisms such as pathogens and herbivores, and they also cannot include all of the abiotic factors of the sites, such as local microclimatic conditions. As a result, field reciprocal transplant experiments regarding local adaptation often produce different results from those in common gardens [7], [19]. Field experiments are expected to provide more realistic insight into the issue of local adaptation than common garden experiments [19].

We studied the role of arbuscular mycorrhizal fungi in adaptations of *Aster amellus* to local soil conditions. We conducted this study in two regions that differ significantly in their soil nutrient content [20], [21]. A previous study indicated that this species is an obligate mycotrophic herb with high dependency upon mycorrhiza because all plants in the non-inoculated treatment died within a few months [20]. We expected to find local adaptation in *A. amellus* because its populations are genetically differentiated [21]. Furthermore, we found some evidence of local adaptation after two years in previous experiments with this species [21], [22]. However, local adaptation was found only in the seedling stage and differed among populations [21]. We were interested in whether local adaptation could be detected after five years in the field and if this adaptation was influenced by the association of the plant with mycorrhizal fungi. We selected three sites in each of two regions with diploid populations of *A. amellus*. We collected seeds in the field and planted juvenile plants pre-grown from the seeds from each population into each population. We monitored plant growth in the field for five years. After that, we determined the aboveground dry biomass and the percentage of mycorrhizal colonisation in the roots. We collected data in only four out of the six populations (two in each region).

We asked the following questions: 1. Does the mycorrhizal colonisation of *A. amellus* plants differ among populations in the field? 2. Is there any evidence of local adaptation in *A. amellus* after five years in the field? 3. Do the differences in root colonisation between plants from different regions and populations match the patterns of plant growth in the field?

## Materials and Methods

### Ethics statements

The administration of Czech Karst, a protected landscape area, permitted us to work with the endangered herb *Aster amellus* in the protected area and in the other locations. No specific permissions were required for field work in the Ceske Stredohori region because the sites were not privately owned or protected in any way.

### Study species and sites

*Aster amellus* L. (Asteraceae) is an endangered, perennial plant growing in dry grasslands in Europe and Asia [23]. *A. amellus* occurs as a diploid and hexaploid cytotype in the Czech Republic [23]. The two cytotypes occur in close proximity, but they never form mixed populations in the Czech Republic [24]. We used only diploid populations for this study.

We conducted this study in two regions in the Czech Republic that differ in their geological bedrock, vegetation composition and soil conditions. The first region, Ceske Stredohori (region S), is characterised by marl bedrock and vegetation belonging to the *Bromion* community [25]. The second region, Czech Karst (region K), is characterised by limestone bedrock and oak-hornbeam forests of the association *Querco pubescenti-petraeae* (thermophile oak wood) [26].

We selected three sites with populations of *Aster amellus* in each of the two regions (Table 1). The distance between the two regions was approximately 70 km and the distances between sites within regions ranged from 2.5 to 17 km. We measured the soil conditions in all sites. We collected five samples of the upper soil layer at each site. We transferred them to the laboratory, air-dried them, sieved them through 2 mm mesh and homogenised them. In the laboratory, we determined the actual pH and analysed the concentrations of Ca^2+^, Mg^2+^, K^+^ following Moore and Chapman [27] and evaluated the available P using the photometric method [28], the total N and total C content following Ehrenberger and Gorbach [29] and the organic C and carbonate content using the volumetric method according to ISO-Standard (10693). We tested for differences in soil properties among sites and regions with a hierarchical analysis of variance (site nested within region) (Table 1). The nutrient content of the soil was generally low in all sites, but the sites in region K had significantly more potassium, nitrogen and organic carbon contents but significantly lower carbonate content than the sites in region S (Table 1).

**Table 1:** Location and soil properties of the studied sites with *Aster amellus* populations in the Czech Republic. Region K is Czech Karst, region S is Ceske Stredohori.

Site	Region	Longitude E	Latitude N	pH (H_2_O)	Ca^2+^(mg/g)	Mg^2+^(μg/g)	K^+^(μg/g)	P(μg/g)	Total N (%)	Corg (%)	Carbonates (%)
K1 - Koda	K	14° 07′ 29″	49° 56′ 01″	5.44	4.68	191.04	271.46	6.70	0.44	6.53	0.02
K2- Karlík	K	14° 15′ 02″	49° 56′ 52″	6.20	4.98	737.73	217.49	11.13	0.46	6.48	0.03
K3 - Lochkov	K	14° 20′ 16″	49° 59′ 56″	7.74	10.84	299.38	282.86	18.80	0.53	7.26	2.96
S1- Malíč	S	14° 05′ 16″	50° 32′ 24″	7.86	8.25	74.74	167.67	7.54	0.21	1.99	6.16
S2 - Holý vrch	S	14° 13′ 49″	50° 31′ 41″	7.84	9.71	80.07	180.42	8.47	0.26	3.72	5.15
S3 - Encovany	S	14° 15′ 33″	50° 31′ 46″	8.05	9.36	37.32	82.89	7.74	0.12	1.89	6.39
Significant differences between regions							K>S		K>S	K>S	K<S

Significant differences (p<0.05) between regions are indicated in the table (df  = 1, df error  = 4). All soil propereties differed significantly (p<0.05) among sites within regions (df  = 4, df error  = 24).

Five arbuscular mycorrhizal fungi species were found in the soil from the studied sites (*Glomus mosseae, G. intraradices, G. microaggregatum, G. etunicatum, and G. constrictum*; H. Pánková and K. Krak, unpublished). Previous garden experiments and field observations indicated that the percentage of mycorrhizal colonisation differs between populations of *A. amellus*, has a genetic basis and is likely driven by the nutrient content in the soil of the natural populations [10], [20]. Mycorrhizal colonisation was found to be significantly lower in region K than in region S [20].

### Reciprocal transplant experiment

We collected seeds of *A. amellus* from each of the six sites in September 2003. In February 2004, we sowed c. 300 seeds from each site in plastic trays with garden substrate and placed them in the greenhouse at 10 °C with natural light conditions. In April 2004, we transplanted the seedlings into multipot trays with pots of 3×3 cm and kept them in a common garden. In October 2004, we transplanted small juvenile plants into the field. We conducted a full reciprocal transplant experiment, so that plants from each site were planted at each site. Therefore, we distinguish the target site (where the plants were planted experimentally) and the site of origin (where the plants had come from). At each target site, we used a randomised block design and planted 150 juvenile plants (6 sites of origin ×25 replicates) into five randomly placed rows. The distance between the two nearest plants in a row was 10 cm. We marked each plant with a nail and a metal label with a number for later recovery with a metal detector. We watered the plants immediately after planting.

Because this perennial plant grows very slowly in the field, we harvested the experiment after five years, in October 2009. We recorded the survival percentage, the number of leaves and the number of flowers in the field. Because few were flowering (<1%), we could not analyse plant reproduction data in this study. We extracted each plant with large soil monoliths (approximately 15 cm in diameter and 10 cm deep) and harvested both the aboveground biomass and the roots for laboratory analyses. In the laboratory, we weighed the aboveground biomass after drying it to a constant weight at 70 °C for 72 hours. We carefully washed the roots from the soil, cut them into segments and stained them with 0.05% Trypan blue in lactoglycerol [30]. We quantified the mycorrhizal colonisation in the roots using the modified segment method [31] under a compound microscope at 200× magnification.

Because of soil erosion at two sites and the subsequent damage of large parts of the experiment, we collected data from all six sites of origin only in four target sites (two in each region; K1, K3, S1, S2). In those four sites, we planted 600 plants in total. However, 53 plants were destroyed by animals at the beginning of the experiment. Therefore, we calculated the percent survival out of 547 plants. The mean survival percentage was 51% (Table 2), which is quite high after five years in the field. Thus, the data on plant size (number of leaves and aboveground biomass) were collected for 281 plants (4–22 plants per target site and site of origin). Finally, mycorrhizal colonisation was assessed for 191 plants (68% of the surviving plants) because of the poor quality of the roots in the remaining plants (roots that were too thick or too short).

**Table 2:** Performance (mean and SE) of plants from six sites of origin (So) at four target sites (St) in a reciprocal transplant experiment with *Aster amellus* after five years; sites K1–K3 are in region K, sites S1–S3 are in region S: a) survival, b) number of leaves, c) aboveground biomass, and d) mycorrhizal colonisation.

Region	Region K (Czech Karst)			Region S (Ceske Stredohori)			Total
So	K1	K2	K3	S1	S2	S3	
a) Survival (%)							
St K1	25.00 (9.93)	35.00 (10.94)	25.00 (9.93)	21.05 (9.61)	38.89 (11.82)	21.05 (9.61)	26.72 (4.13)
St K3	72.73 (9.72)	66.67 (9.83)	40.00 (10.00)	45.83 (10.39)	54.17 (10.39)	52.38 (11.17)	55.00 (4.22)
St S1	64.00 (9.80)	58.33 (10.28)	48.00 (10.20)	56.00 (10.13)	25.00 (9.03)	24.00 (8.72)	45.95 (4.11)
St S2	69.57 (9.81)	69.57 (9.81)	75.00 (9.03)	76.00 (8.72)	88.00 (6.63)	56.52 (10.57)	72.73 (3.74)
Total	58.89 (5.22)	58.24 (5.20)	46.81 (5.17)	51.61 (5.21)	52.75 (5.26)	38.64 (5.22)	51.37 (2.14)
b) Number of leaves							
St K1	2.40 (0.60)	5.00 (3.41)	2.75 (0.75)	3.60 (0.98)	2.00 (0.38)	3.00 (1.30)	3.13 (0.68)
St K3	5.31 (1.03)	3.88 (1.00)	4.80 (0.83)	4.91 (0.86)	6.15 (1.37)	4.55 (0.65)	4.92 (0.42)
St S1	3.06 (0.42)	2.86 (0.31)	3.83 (0.69)	3.71 (0.59)	3.00 (0.58)	5.50 (1.23)	3.50 (0.25)
St S2	4.63 (0.71)	4.06 (0.41)	6.33 (0.80)	8.00 (1.21)	8.18 (1.41)	6.31 (0.61)	6.41 (0.44)
Total	4.15 (0.42)	3.79 (0.50)	4.98 (0.46)	5.63 (0.60)	6.08 (0.81)	5.14 (0.44)	4.93 (0.23)
c) Aboveground biomass (mg)							
St K1	61.60 (14.62)	90.50 (46.82)	60.00 (20.26)	102.00 (13.77)	67.20 (26.09)	118.20 (11.30)	83.66 (11.48)
St K3	213.13 (57.59)	166.06 (37.68)	199.50 (41.96)	209.30 (37.44)	224.08 (43.89)	328.45 (124.77)	219.49 (25.19)
St S1	130.67 (40.07)	84.71 (26.95)	59.83 (42.68)	98.67 (35.47)	50.80 (7.63)	67.00 (29.24)	90.00 (14.61)
St S2	142.00 (37.30)	172.88 (33.20)	151.83 (30.75)	200.61 (30.04)	186.95 (35.33)	150.31 (33.22)	169.06 (13.56)
Total	154.24 (24.86)	137.54 (18.30)	140.18 (20.82)	170.73 (18.99)	168.42 (23.02)	190.97 (44.65)	158.65 (10.18)
d) Mycorrhizal colonisation (%)							
St K1	26.98 (7.39)	46.18 (8.14)	67.41 (14.37)	52.44 (18.25)	65.83 (8.96)	56.29 (14.80)	51.79 (5.07)
St K3	70.03 (5.73)	72.72 (5.77)	66.47 (8.54)	91.96 (1.71)	91.13 (2.25)	78.85 (4.54)	78.41 (2.47)
St S1	77.59 (6.99)	76.66 (4.23)	82.85 (3.49)	88.48 (2.63)	92.13 (3.00)	89.80 (4.15)	83.69 (1.91)
St S2	70.45 (5.84)	79.08 (6.31)	68.85 (7.92)	91.15 (2.95)	85.70 (5.13)	93.48 (3.10)	80.95 (2.54)
Total	65.94 (4.15)	71.27 (3.41)	72.11 (3.96)	85.76 (3.20)	85.49 (2.78)	81.54 (4.13)	76.57 (1.57)

All of the values are the untransformed, original data.

### Data analysis

To analyse the data in the reciprocal transplant experiment, we used an analysis of variance for normally distributed variables (mycorrhizal colonisation, the number of leaves and aboveground biomass) and an analysis of deviance for binomial variables (survival). We log-transformed both the number of leaves and the aboveground biomass to fit the assumptions of normality. We distinguished the target region and site (where the plants were planted experimentally) and the region and site of origin (where the plants had come from) of the transplanted plants. We tested the target region against target site and target site against block (five blocks, represented by rows in each site; Table 3). Similarly, we tested region of origin against site of origin and site of origin against the target site-by-site of origin interaction (Table 3). Then, we tested the interaction between target region and region of origin against the interaction between target site and site of origin, which was tested against residuals (Table 3). A significant interaction between target region and region of origin would indicate local adaptation at the regional scale. Similarly, a significant target site-by-site of origin interaction would indicate local adaptation (or maladaptation) at the smaller scale. We also tested for local versus foreign contrast, as was performed in [21], but it was not significant and therefore we do not show the results. Where the interaction between the target region and region of origin was significant, we tested for the effect of region of origin in each target region separately. We followed the statistical design of the study and tested the region of origin against the site of origin and the site of origin against residuals.

**Table 3:** Summary of the analysis of variance (F values) and deviance (Quasi-F values) for the effects of target region (Rt), target site (St; nested within target region), block (nested within target site), region of origin (Ro), site of origin (So; nested within region of origin) and their interactions on survival percentage (Survival), the logarithm of the number of leaves (Leaves), the logarithm of the aboveground biomass (Biomass) and percentage of mycorrhizal colonisation (Colonisation).

Source of variation	DF	Error	Survival	Leaves	Biomass	Colonisation
			Quasi-F	F value	F value	F value
Rt	1	St	0.73	0.41	0.28	1.22
St	2	Block	6.90**	12.15***	3.83*	6.91**
Block	16	Residuals	2.59***	2.03*	4.98***	4.19***
Ro	1	So	1.46	5.14+	26.08**	49.86**
So	4	St × So	1.45	2.22	0.26	0.56
Rt × Ro	1	St × So	0.43	4.15+	0.40	0.31
St × So	14	Residuals	1.35	0.60	0.80	1.66+
Residual df			507	241	241	151

Error indicates the error term used for each source of variation.

***p<0.001; **p<0.01; *p<0.05; +p<0.1.

To test for the relationship between mycorrhizal colonisation and plant growth, we used a general linear model (GLM). We used the number of leaves and aboveground biomass (both log scale) as dependent variables and the percentage of mycorrhizal colonisation as an independent variable. Because we expect that mycorrhizal colonisation of the plants will differ among sites and regions, we used target region, target site, region of origin and site of origin as covariates in the analyses. All tests were performed in the statistical program S-plus 6.2 (Insightful Corp., Seattle, Washington, U.S.A.).

## Results

**Figure 1:**
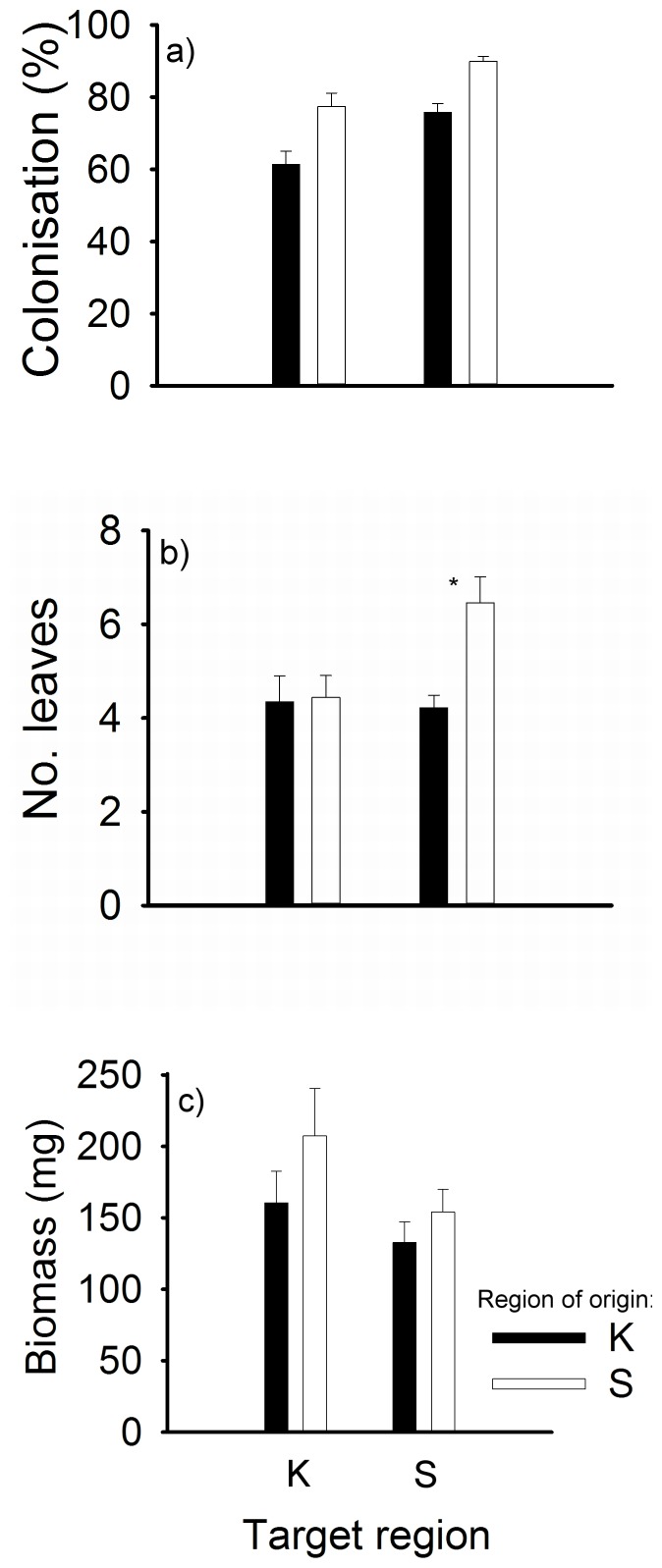
Differences in mycorrhizal colonisation and plant growth parameters between regions of origin and target regions. a) Mycorrhizal colonisation, b) The number of leaves, and c) Aboveground biomass. Region K is Czech Karst, region S is Ceske Stredohori. Values represent the means ± 1 SE. To allow ecological evaluation of the data, we present untransformed data for the number of leaves and aboveground biomass. Asterisk (*) denotes significant effect (p<0.05) of region of origin within a target region.

Both target site and block within the target sites significantly affected mycorrhizal colonisation, survival percentage, the number of leaves and aboveground biomass, although the target region did not (Table 3). The region of origin significantly affected mycorrhizal colonisation and the aboveground biomass, and it also affected the number of leaves with marginal significance (Table 3). Specifically, plants originating from region S had higher mycorrhizal colonisation, higher aboveground biomass and more leaves than plants originating from region K (Table 2; Fig. 1).

There was a marginally significant difference in mycorrhizal colonisation between the sites of origin within target sites (St × So interaction; Table 3). The number of leaves differed slightly between regions of origin within target regions (Rt × Ro interaction; Table 3). In region S, plants from the local region had significantly more leaves than plants from the foreign region (F_1, 4_ = 11.93, p = 0.026), while the number of leaves did not differ significantly between the regions of origin in region K (F_1, 4_ = 0.72, p = 0.44; Fig. 1b).

After adjusting for the effects of target site and site of origin, we found a significant positive relationship between mycorrhizal colonisation and aboveground biomass (GLM; log scale; r = 0.16, p = 0.013, df error  = 169) but not the number of leaves (GLM; log scale; r = 0.03, p = 0.703, df error  = 181).

## Discussion

### Mycorrhizal colonisation

Through a reciprocal transplant experiment in the field, we demonstrated that mycorrhizal colonisation has a genetic basis and varies among regions of origin. In both regions, plants from region S had higher mycorrhizal colonisation than plants from region K. This finding agrees with previous greenhouse experiments using *A. amellus* [10], [20]. Similarly, a genetic basis of mycorrhizal colonisation was found in a greenhouse experiment with *Plantago lanceolata*, in which the percentage of mycorrhizal colonisation in the experiment corresponded to the field colonisation [9]. In contrast, another study using *Andropogon gerardii* suggested that the plants can change their percentage of root colonisation depending on soil nutrient content [8]. Overall, this is the first study that showed that mycorrhizal colonisation has a genetic basis using a field experiment. Furthermore, we demonstrated that the predetermination to low and high percentage of root colonisation persisted after five years of growth in foreign conditions.

### Local adaptation

We found no evidence of local adaptation in terms of plant growth in this experiment at the population or regional level, i.e., no significant interaction between target site and site of origin or between target region and region of origin. There was only a marginally significant target region-by-region of origin interaction in the number of leaves. However, the number of leaves is not an appropriate measure of fitness in the absence of an effect on plant biomass. In long-lived perennials, belowground biomass may also be an important indicator of plant fitness, as is aboveground biomass. However, it is not possible to completely excavate belowground biomass in the field. Therefore, we could not estimate this important parameter in our study. Nevertheless, some studies have shown a strong correlation between above- and belowground biomass in garden experiments (e-g. [7], [10]).

Generally, plants from region S outperformed plants from region K in both target regions. In a previous study that compared the performance of *A. amellus* in the same reciprocal transplant experiments after two years, we found some evidence of local adaptation at the population level [21]. However, local adaptation was found only in the seedling stage and differed among populations [21]. As in the current study, no local adaptation was found in the transplant experiment for juvenile plants after two years [21].

The absence of local adaptation has also been reported in other plant species, such as *Carlina vulgaris* [32], *Crotalaria pallida* [33], *Stipa capillata* [34] and *Helleborus foetidus* [35]. Each of these studies provided different explanations for the lack of local adaptation. The absence of local adaptation in *Carlina vulgaris* at the local spatial scale, despite strong genetic differentiation between populations, was explained by potential adaptation to other factors (such as pathogens or mutualistic organisms) that were not included in the study [32]. The lack of regional adaptation in the legume plant *Crotalaria pallida* was attributed to the unstable population structure of its herbivore [33]. Furthermore, the absence of local adaptation of *Stipa capillata* to their soil biota from Europe and Asia could be due to the similar composition of rhizosphere biota across populations and regions, sufficient gene flow, selection in favour of plasticity, or a functional redundancy among different soil biota [34]. Moreover, the lack of evidence for local adaptation in *Helleborus foetidus* could be caused by a congruency in selective pressures exerted by different soil environments on seedling emergence and survival [35].

In our study, plant aboveground biomass and mycorrhizal colonisation exhibited corresponding differences between the two target regions and the regions of origin. Because all of the studied sites are rather nutrient-poor, arbuscular mycorrhiza should have beneficial effects on the growth of *Aster amellus* according to the coadaptation model [12], [14]. A previous experiment, where the percentage of mycorrhizal colonisation was manipulated, demonstrated the positive effects of mycorrhizal colonisation on the growth of *A. amellus* (Pánková et al., unpubl.), a finding that supports the coadaptation model. In the present study, we did not find any significant relationship between mycorrhizal colonisation and the number of leaves, and the positive relationship between mycorrhizal colonisation and aboveground biomass was quite weak (r = 0.16). Nevertheless, we tested for the relationship after adjusting for the effects of target site and site of origin. Therefore, the weak relationship could be explained by a low variation in mycorrhizal colonisation within natural populations [20]. As mycorrhizal colonisation differs significantly between the two regions of origin, the main effect of mycorrhizal colonisation on the growth of *A. amellus* was pronounced at the regional scale.

The genetic differences in mycorrhizal colonisation between plants from the two regions under study indicate that the percentage of mycorrhizal colonisation in the roots is an adaptive trait. Particularly, mycorrhizal colonisation was higher in the plants from region with lower soil nutrient content (region S). It is likely that higher mycorrhizal colonisation is the reason why the plants from region S outperformed the plants from region K in both target regions. This implies that the plants from region K are maladapted via their low inherent mycorrhizal colonisation levels.

### Conclusions

Our study showed significant differences in mycorrhizal colonisation between regions of origin after five years in the field, indicating that the percentage of mycorrhizal colonisation of the roots has a genetic basis. This study confirms our previous findings from pot experiments using the same plant species *A. amellus* [10], [20]. Overall, we did not find any evidence of local adaptation in terms of aboveground biomass in *A. amellus* in this study. Instead, plants from region S outperformed plants from region K in both target regions. Similarly, plants from region S showed higher mycorrhizal colonisation in all cases, which was driven by the lower nutrient content in the soil from this region. Thus, the pattern of plant aboveground biomass corresponded with the differences in mycorrhizal colonisation between the two regions. Higher mycorrhizal colonisation in the plants from region with lower soil nutrient content (region S) in both target regions indicates that mycorrhizal colonisation is an adaptive trait. However, lower aboveground biomass in the plants with lower mycorrhizal colonisation suggests that the plants from region K are in fact maladapted by their low inherent mycorrhizal colonization levels. We conclude that including mycorrhizal symbiosis in local adaptation studies may increase our understanding of the mechanisms by which plants are adapting to their environment.

### Data availability statement

The data are available as a Supporting Information file Table S1.

## Supporting Information

Table S1 **Data from: Mycorrhizal symbiosis and local adaptation in** ***Aster amellus*** **: a field transplant experiment.**
(XLS)
